# Brain Phospholipid Precursors Administered Post-Injury Reduce Tissue Damage and Improve Neurological Outcome in Experimental Traumatic Brain Injury

**DOI:** 10.1089/neu.2017.5579

**Published:** 2018-12-14

**Authors:** Orli Thau-Zuchman, Rita N. Gomes, Simon C. Dyall, Meirion Davies, John V. Priestley, Martine Groenendijk, Martijn C. De Wilde, Jordi L. Tremoleda, Adina T. Michael-Titus

**Affiliations:** ^1^ Centre for Neuroscience and Trauma, The Blizard Institute, Barts and The London School of Medicine and Dentistry, Queen Mary University of London, London, United Kingdom.; ^2^ Nutricia Research–Nutricia Advanced Medical Nutrition, Utrecht, The Netherlands.; ^3^ Bournemouth University, Royal London House, Bournemouth, United Kingdom.

**Keywords:** brain phospholipids, functional improvement, medical multi-nutrient, neuroplasticity, neuroprotection, traumatic brain injury

## Abstract

Traumatic brain injury (TBI) leads to cellular loss, destabilization of membranes, disruption of synapses and altered brain connectivity, and increased risk of neurodegenerative disease. A significant and long-lasting decrease in phospholipids (PLs), essential membrane constituents, has recently been reported in plasma and brain tissue, in human and experimental TBI. We hypothesized that supporting PL synthesis post-injury could improve outcome post-TBI. We tested this hypothesis using a multi-nutrient combination designed to support the biosynthesis of PLs and available for clinical use. The multi-nutrient, Fortasyn^®^ Connect (FC), contains polyunsaturated omega-3 fatty acids, choline, uridine, vitamins, cofactors required for PL biosynthesis, and has been shown to have significant beneficial effects in early Alzheimer's disease. Male C57BL/6 mice received a controlled cortical impact injury and then were fed a control diet or a diet enriched with FC for 70 days. FC led to a significantly improved sensorimotor outcome and cognition, reduced lesion size and oligodendrocyte loss, and it restored myelin. It reversed the loss of the synaptic protein synaptophysin and decreased levels of the axon growth inhibitor, Nogo-A, thus creating a permissive environment. It decreased microglia activation and the rise in ß-amyloid precursor protein and restored the depressed neurogenesis. The effects of this medical multi-nutrient suggest that support of PL biosynthesis post-TBI, a new treatment paradigm, has significant therapeutic potential in this neurological condition for which there is no satisfactory treatment. The multi-nutrient tested has been used in dementia patients and is safe and well tolerated, which would enable rapid clinical exploration in TBI.

## Introduction

Traumatic brain injury (TBI) is a leading cause of death and disability^1–3^ and survivors suffer from cognitive and psychological disorders. The deficits post-TBI result from multiple neurochemical and metabolic events,^4^ leading to neuronal loss, dendritic, axonal, and synaptic changes,^5^ and white matter abnormalities.^6–8^ TBI increases the risk of neurodegenerative diseases such as Alzheimer's disease (AD)^9^ and Parkinson's disease.^10^

Experimental and human TBI observations report significant synaptic alterations.^11–15^ Brain connectivity is disrupted in TBI patients and this is related to cognitive dysfunction.^16,17^ Phospholipids (PLs) are key to the structure of membranes, and TBI triggers significant changes in PL. A decrease in brain PL, subsequent to phospholipase activation, was reported in rat controlled cortical impact injury (CCI) 4 days post-TBI and was still detected at 35 days.^18^ Homayoun and colleagues reported changes in free fatty acids, a consequence of PL degradation, as early as 30 min post-CCI.^19^ Changes in PL have been reported in cerebrospinal fluid and brain tissue of TBI patients and in mice post-CCI.^20,21^ Plasma lipidomics shows lower levels of PL in chronic TBI in humans.^22^ This raises the question whether PL breakdown could become a treatment target in TBI.

Membranes contain high levels of PL^23,24^; the “Kennedy pathway,” a series of biochemical reactions that represent the main mechanism by which mammalian cells synthesize PL, such as phosphatidylcholine (PC) and phosphatidylethanolamine (PE), uses precursors such as uridine, choline, and polyunsaturated fatty acids (PUFAs) such as the omega-3 PUFA, docosahexaenoic acid (DHA).^25,26^ Lower levels of PUFA and a decreased ratio DHA/arachidonic acid (AA) in PL were detected in brain and plasma in mice post-CCI.^21^ Low plasma choline was reported in TBI patients.^27^ This suggests that TBI creates a need for sustained support of PL biosynthesis, which could be addressed by providing precursors.

Studies on PL biosynthesis^28,29^ have led to the development of a medical multi-nutrient, Fortasyn^®^ Connect (FC), designed to support PL formation. FC contains DHA and also the omega-3 PUFA, eicosapentaenoic acid (EPA), and uridine monophosphate (UMP), choline, folic acid, vitamins B12, B6, C, and E, and selenium, required for PL biosynthesis. FC supports synaptogenesis^29–31^ and improves learning and memory.^30,32^ FC protects against β-amyloid toxicity,^33^ and the beneficial impact of FC on memory impairment in early AD is supported by several clinical trials.^34–36^ Given that TBI leads to a decrease in PL levels in brain and plasma, we hypothesized that FC, by providing PL precursors, could have significant beneficial effects in TBI.

The aim was to test the effect of an FC-supplemented diet in mice with CCI injury, a model that reproduces clinically relevant neurobehavioral changes,^37^ and where PL changes have been documented over a long period post-injury.^21^ Microglial activation, hippocampal neurodegeneration and myelin loss persist up to 1 year in CCI.^38^ CCI studies have reported protracted increases in lesion volume^39^ and impairment of spatial learning and memory.^40^ We hypothesized that administration of FC post-TBI could improve neurological outcome and counter tissue loss.

## Methods

### Animals

Adult 10- to 12-week-old male C57BL/6 mice, 22–27 g (Charles River Laboratories, Harlow, UK; http://www.criver.com/files/pdfs/rms/c57bl6/rm_rm_d_c57bl6n_mouse.aspx), were used, housed in groups of 4 in cages provided with enrichment objects, in a 12-h light/dark cycle, with diet and water *ad libitum*. Food intake and body weight were monitored daily. All animal procedures were carried out under a Project Licence approved by the Animal Welfare and Ethical Review Body, at Queen Mary University of London and the UK Home Office, in accord with the EU Directive 2010/63/EU.

### Controlled cortical impact model

A CCI TBI model was used.^41^ After a 1-week acclimatization period, mice were anesthetized using ketamine (50 mg/kg) and medetomidine (0.5 mg/kg), administered intraperitoneally (i.p.). Mice were placed in a stereotaxic frame and a midline longitudinal incision was performed to expose the skull. A right lateral craniotomy was carried out using a pneumatic drill, 2.0 mm behind bregma, and 2.5 mm lateral to the midline. CCI injury was induced using the following settings: a 3-mm impactor tip with a speed of 3 m/s, a depth of 2.2 mm, and a dwell time of 100 ms, applied using the PCI3000 Precision Cortical Impactor™ (Hatteras Instruments, Inc., Cary, NC). A control group underwent craniotomy only. Post-injury, the skull flap was placed back and the skin was sutured. Mice were allowed to recover in an incubator (37°C) until they were fully awake and active. Buprenorphine (0.05 mg/kg), administered subcutaneously, was used pre-operatively for pre-emptive analgesia and post-operatively every 12 h for 3 days post-TBI.

### Dietary supplementation

Post-CCI, mice were randomized into two groups and fed with a control diet (CCI-Control; *n* = 10) or with a FC multi-nutrient diet (CCI-FC; *n* = 10) for 70 days (detailed composition in Table 1). The craniotomy group were fed with control diet (craniotomy; *n* = 10), and there was no craniotomy-FC group, because the emphasis of the study was on the brain injury component. The choice of the dose used in this study was based on a previous study carried out in our group, in which we tested several dose levels.^42^ The diets were formulated by Nutricia Research (Utrecht, The Netherlands) and manufactured by Ssniff (Soest, Germany), stored at −20°C to prevent lipid peroxidation, and fresh diet was given daily. Diet stability under these conditions has been confirmed by the producers of the diet. No significant differences were observed in the daily food intake and body weight (Supplementary Fig. 1) (see online supplementary material at http://www.liebertpub.com) between groups.

**Table 1.** Compositions of the Experimental Diets (g/100 g)

*Ingredient*	*Control*	*FC*
Corn starch	35.57	31.31
Caseine (>85% protein)	14.00	14.00
Corn dextrine	15.50	15.50
Sucrose	10.00	10.00
Dextrose	10.00	10.00
Fiber	5.00	5.00
Mineral mix (AIN-93M-MX)	3.50	3.50
Vitamin mix (AIN-93-VX)	1.00	1.00
Choline bitartrate (41.1% choline)	0.250	0.250
l-cystine	0.180	0.180
Tert-butylhydroquinone	0.0008	0.0008
Oil blends		
Soy oil	1.900	—
Coconut oil	0.900	0.100
Corn oil	2.200	0.100
DHA25 oil	—	4.500
EPA28/12	—	0.300
Other additions		
Pyridoxine-HCL	—	0.00529
Folic acid (90%)	—	0.00111
Cyanocobalamin (0.1% in mannitol)	—	0.00650
Ascorbic acid (100% pure)	—	0.24000
dl-α-tocopheryl acetate (500 IU/g)	—	0.70500
UMP disodium (24% H_2_O)	—	1.50000
Choline chloride (74.576%)	—	0.67046
Soy lecithine (Emulpur)	—	1.13205
Total phospholipids	(—)	(0.8717)
Phosphatidylcholine	(—)	(0.2264)
Phosphatidylinositol	(—)	(0.1585)
Phosphatidylethanolamine	(—)	(0.1472)
Sodium selenite (46% min)	—	0.00036
**Total**	100.0	100.0

*Fatty acid profile*	*Control*	*FC*
C-4:0	0.000	0.000
C-6:0	0.004	0.001
C-8:0	0.064	0.007
C-10:0	0.052	0.006
C-12:0	0.395	0.046
C-14:0	0.155	0.191
C-14:1 n5	0.000	0.000
C-15:0	0.000	0.035
C-16:0	0.492	0.795
C-16:1 n7	0.004	0.251
C-17:0	0.000	0.042
C-18:0	0.136	0.222
C-18:1 n9	1.041	0.656
C-18:2 n6	2.181	0.158
C-18:3 n3	0.107	0.038
C-18:3 n6	0.000	0.006
C-18:4 n3	0.000	0.001
C-20:0	0.020	0.018
C-20:1 n9	0.010	0.097
C-20:2 n6	0.000	0.035
C-20:3 n6	0.000	0.008
C-20:4 n3	0.000	0.000
C-20:4 n6	0.000	0.092
C-20:5 n3	0.000	0.433
C-22:0	0.009	0.011
C-22:1 n9	0.000	0.012
C-22:4 n6	0.000	0.017
C-22:5 n3	0.000	0.074
C-22:6 n3	0.000	1.117
C-24:0	0.000	0.008
C-24:1 n9	0.000	0.014
Total FA	4.771	4.771
SAT FA	1.327	1.382
MUFA	1.055	1.030
PUFA	2.288	1.979
Other FA	0.101	0.379
MCT	0.515	0.060
Tot n6	2.181	0.316
Tot n3	0.107	1.663
n6/n3	20.298	0.190

UMP, uridine monophosphate; FC, Fortasyn® Connect; FA, fatty acid; SAT FA, saturated fatty acid; MUFA, monounsaturated fatty acid; PUFA, polyunsaturated fatty acid; MCT, medium-chain triglyceride.

### Experimental design and behavioral testing

**FIG. 1.**
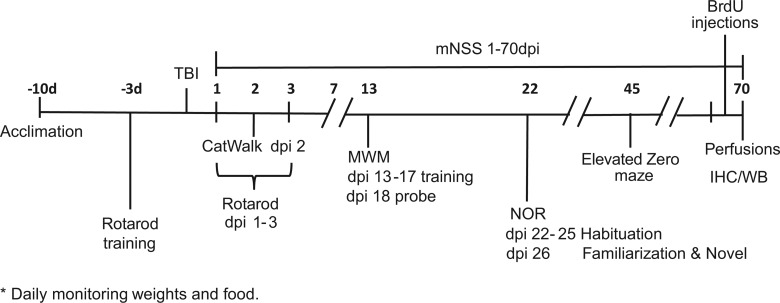
Experimental design. Behavioral testing was performed for 70 days post-injury (dpi). A controlled cortical impact (CCI) or a control craniotomy (sham injury) were induced in adult male mice. Animals were divided into three experimental groups (craniotomy-control, CCI-Control, and CCI-FC). All mice were tested for motor and cognitive impairments on all behavioral tests. Mice were trained for 3 consecutive days for the Rotarod test pre-injury. Throughout the study (1–70 dpi), mice were tested for mNSS every other day on the first week and once a week thereafter, Rotarod (1–3 dpi), CatWalk (2 dpi), MWM (13–18 dpi), novel object recognition (NOR; 22–26 dpi), and elevated zero maze (EZM; 45 dpi). A week before the end of the study mice were injected twice a day with BrdU. Mice were monitored daily for weight and food consumption. On 70 dpi, mice underwent perfusion for immunohistochemistry (IHC) analysis or were decapitated and brains were quickly removed and snap frozen for western blot (WB) analysis. Seventy-day plasma and cerebellar tissue samples were used for lipid analysis. BrdU, bromodeoxyuridine; FC, Fortasyn^®^ Connect; MWM, Morris water maze; mNSS, Modified Neurological Severity Score; TBI, traumatic brain injury.

Testing at various days post-injury (dpi) is summarized in Figure 1. Sample size was calculated using power analysis (https://eda.nc3rs.org.uk/eda/) for pair-wise post-hoc comparisons after analysis of variance (ANOVA), to a statistical power of 90%, with a significance level α = 0.05 to detect a 25% and 20% relative difference in sensorimotor behavioral scoring (Modified Neurological Severity Score; mNSS), lesion size, and histopathology (glial responses, cell proliferation, and white matter), as experimental primary and secondary outcomes. All the behavior (the primary study endpoint) was assessed in “blind,” with the researcher unaware of the treatment, in accord with ARRIVE (Animal Research: Reporting of In Vivo Experiments) guidelines.

### Modified Neurological Severity Score

The mNSS was used to evaluate motor ability, balance, and alertness, using a scoring system based on the ability to perform 10 tasks that evaluate motor ability, balance, and alertness (Supplementary Fig. 2) (see online supplementary material at http://www.liebertpub.com). During the first week, testing was performed every other day, then once a week, until the end of experiment. A point was given for failure to perform a task. Tissue pathology correlates well with impairment scores and with degree of brain edema.^43,44^ The first mNSS was obtained 24 h post-TBI.

### Rotarod

The Rotarod test (Ugo Basile SRL, Gemonio, Italy; 3 cm in diameter) was used for the evaluation of motor coordination and balance and was carried out between days 1 and 3 post-CCI. Pre-surgery, mice were trained on the Rotarod for 3 consecutive days. The first two trials were 60 sec each, at a speed of 3 rpm, followed by a single trial at accelerating speed (3–20 rpm over 300 sec), with intervals of at least 25 min of rest. The rest of the training days consisted of one trial at a constant speed of 3 rpm followed by two accelerating trials. The latency to fall from the Rotarod was recorded. The average of all accelerating phase scores was considered as the baseline (pre-injury score). Mice were tested during days 1–3 post-injury, in three trials a day, using the accelerating mode.

### CatWalk

The CatWalk is a system for a quantitative automated assessment of gait. It consists of an enclosed walkway, a camera, and recording and analysis software (Noldus/Tracksys Ltd, Nottingham, UK; RRID:SCR_004074). Dynamic gait parameters were analyzed on day 2 post-CCI. Each mouse was placed individually in the walkway, which consists of a glass plate (100 × 15 × 0.6 cm) surrounded by two black Plexiglas walls, spaced 8 cm apart. The mouse was allowed to walk freely and traverse from one side to the other of the walkway. Two infrared beams spaced 90 cm apart were used to detect the arrival of the mouse and control the start and end of data acquisition. Recordings were carried out in a dark room. After each trial, the walkway was cleaned with 1% acetic acid for odor neutralization. CatWalk XT 7.0 software (Noldus) was used to analyze the data.

### Morris water maze

The Morris water maze test (MWM) was used to assess memory deficits associated with spatial learning^45^ between days 13 and 18 post-CCI. A 100-cm-diameter pool filled with opaque water at 23°C was placed inside a white tent, ensuring light uniformity, with four visible cues hung 10 cm from the pool walls and an 11-cm diameter Plexiglas resting platform submerged 0.5 cm below the water level. Swimming performance (e.g., path, distance, speed, and latency) was tracked using software (ANY-maze, Smart; Bioseb, Vitrolles, France; RRID:SCR_014289). A learning period of 5 consecutive days (days 13–17 post-injury) and a probe trial on day 6 (day 18 post-injury) were used. During the learning period, each mouse was subjected to four trials a day, in the pool divided into four virtual quadrants. The position of the platform was constant throughout the training session, whereas the starting position on each of the four training trials was changed. If a mouse did not find the platform within 60 sec, it was guided to it. After reaching the platform, mice were allowed to stay there for 15 sec. During the probe trial, mice were allowed to swim for 60 sec in the absence of the platform, and the time it took to first enter the quadrant that had previously hosted the platform, was measured.

### Novel Object Recognition

The novel object recognition (NOR) test was used to evaluate recognition memory on day 26 post-CCI. In the habituation phase, on days 22–25 post-injury, animals were exposed to an empty opaque box used as an open field, for 10 min. Twenty-four hours later, in a familiarization phase, each animal was given 20 min to explore two identical objects, placed in the same open field. Four hours later, in the second test phase, animals were exposed to two dissimilar objects placed in the same open field: one familiar object used in the first phase and one novel object. In the test phase, exploration time was 10 min and time spent exploring each of the objects was measured. Performance was tracked with software (ANY-maze, Smart; Bioseb). A recognition index (RI), that is, the time spent investigating the novel object relative to the total object investigation, was calculated as follows: percentage time spent with novel object/time spent with novel and familiar objects. Mice that discriminate between the old and new object should have an RI above 50%.^46^

### Elevated zero maze

An elevated zero maze test (EZM) was used to assess exploratory behavior in an anxiety-provoking environment. Anxiety is expressed by spending more time in the enclosed quadrants. On day 45 after CCI or craniotomy, mice were individually placed in a closed quadrant and allowed to freely explore the maze for 5 min. A camera tracked the animal, and ANY-maze software calculated the time spent in the open quadrants, the head dips (downward movement of the head toward the floor) and stretch-attenuated postures (elongation of the body with the feet remaining in place) from the closed arms, and the total distance travelled during the test.

### Bromodeoxyuridine injections

From day 63 post-injury or craniotomy, and for 7 sequential days, animals received i.p. injections of 5-bromo-2-deoxyuridine (BrdU; 50 mg/kg, twice a day), to assess cell proliferation.

### Histology and immunohistochemistry

At day 70 post-TBI, 5 animals from each group were deeply anesthetized with sodium pentobarbital (50 mg/kg, i.p.; Sagatal; Rhône-Mérieux, Harlow, UK) and received a transcardiac perfusion with phosphate-buffered saline (PBS; 0.01 M, pH 7.4), followed by 4% paraformaldehyde in phosphate buffer (0.1 M, pH 7.4, 4°C). Brains were dissected out and tissue blocks were paraffin-fixed for histology and immunohistochemistry (IHC) analyses. All tissue staining was performed between bregma −1.28 and bregma −2.34, where the lesion was located. All tissue analyses were carried out in “blind” with the researcher unaware of the treatment, in accord with ARRIVE guidelines. Sections (7-μm) were deparaffinized and hydrated through xylene and ethanol baths. Sections were subjected to antigen retrieval (10 mM of citrate buffer, pH 6.0, 30 min at 80°C) and then cooled at room temperature. Tissue was blocked with 5% normal donkey serum in 0.2% Triton X-100 in PBS for an hour, followed by three PBS washes. The following primary antibodies were used (overnight incubation): rat anti-BrdU (for cell proliferation; 1:200; Acris Antibodies GmbH, Herford, Germany; Cat# SM1667PS Lot# RRID:AB_973414); rabbit anti–glial fibrillary acidic protein (GFAP; for astrocytes; 1:800; Dako, Carpinteria, CA; Cat# Z0334 Lot# RRID:AB_10013382); goat anti–ionized calcium binding adaptor molecule 1 (Iba-1; for microglia; 1:800; Wako Chemicals USA, Inc., Richmond, VA; Cat# 019-19741 Lot# RRID:AB_839504) and rabbit anti–translocator protein (TSPO; for glial activation following central nervous system injury and inflammation; 1:100; Abcam, Cambridge, MA; Cat# ab109497 Lot# RRID:AB_10862345); mouse anti–adenomatous polyposis coli (APC; for oligodendroglia; 1:50; Millipore, Billerica, MA; Cat# OP80 Lot# RRID:AB_2057371); rabbit anti–cleaved caspase-3 (Asp175; for apoptosis; Cell Signaling Technology, Danvers, MA; Cat# 9661 also NYUIHC-314, 9661S, 9661L Lot# RRID:AB_2341188); and antidoublecortin (DCX; for immature neurons; 1:100 Millipore; Cat# AB2253 RRID:AB_1586992). The secondary antibodies were Alexa 488 or Alexa 555 (Molecular Probes, Leiden, The Netherlands; 1:200), and Hoechst 33342 stain (Sigma-Aldrich, Gillingham, UK; 1 μg/mL of PBS) was used to visualize nuclei. Slides were mounted and cover-slipped using Vectashield fluorescent mounting medium (H-1000; Vector Laboratories, Burlingame, CA). A subset of representative randomly selected sections across the whole lesion was used for Luxol fast blue (Sigma-Aldrich, UK) myelin staining.

For calculation of lesion size, sections of 7 μm, 200 μm apart, and spanning the entire rostrocaudal extent of the injured cortex were stained with hematoxylin and eosin (H&E). Lesion size was measured with ImageJ software (National Institutes of Health [NIH], Bethesda, MD) and calculated using the equation: the contralateral (nonlesioned) hemisphere size minus the injured hemisphere size and divided by the contralateral hemisphere size.^47^ Results are expressed as a percentage of hemispheric tissue.

### Image capture analysis and processing

Four sections per animal were stained, per antibody. At least 24 fields were captured perilesionally (immediate area around the lesion, except for DCX, which was used to assess the differences in the contralateral hippocampus). Images were viewed at × 40 and photographed using a Zeiss Axioskop 2 microscope (Carl Zeiss, Jena, Germany) with a Hamamatsu camera (C4742-95; Hamamatsu Photonics K.K., Hamamatsu City, Japan). All image capture and quantification were performed blinded. Analyses were done using the ImageJ program and a dedicated script (JVP AutoColourCellCountsRev). Fluorescent signals under different excitation lasers were selected by thresholding and then superimposed on nuclei, for colocalization. A Zeiss LSM 710 confocal microscope was used for further characterization (ZENlite software; Zeiss, Cambridge, UK). Figures were prepared using Illustrator software (Adobe Illustrator CS6; Adobe Systems, San Jose, CA; RRID:SCR_014198). For quantification of microglia morphology, we measured cell size with ImageJ (ImageJ 1.50i; NIH), for a minimum of 20 cells per animal, and 5 animals per group.

### Western blot analysis

Tissue from 5 animals from each group (CCI or craniotomy) was used. At day 70 post-TBI, animals from each group were deeply anesthetized with sodium pentobarbital (50 mg/kg, i.p.; Sagatal; Rhône-Mérieux), and they were decapitated. Brains were removed, and a cube of the right hemisphere around the lesion was dissected using a brain matrix. Tissue was snap-frozen and stored at −80°C. Samples were prepared in radioimunoprecipitation assay lysis buffer (Sigma-Aldrich) complete with Protease Inhibitor Cocktail (Sigma-Aldrich) and sonicated, then centrifuged (10,000*g*, 10 min, 4°C), and the supernatant was taken. Protein concentrations were determined using the Bradford assay. Equal amounts of protein (50 μg) were mixed with NuPAGE^®^ LDS sample buffer (Thermo Fisher Scientific, Waltham, MA) and dithiothreitol and boiled (95°C, 10 min), then separated using Mini-Protean TGX Gels, 10% (Bio-Rad, Watford, UK), and electro-transferred onto polyvinylidene difluoride membranes (BioTrace Medical Inc, Menlo Park, CA). Membranes were blocked in 5% nonfat dry milk in Tris-buffered saline (TBS; pH 7.4), with 0.1% Tween-20 (TBS/Tween) for 1 h at room temperature. The primary antibodies used were: mouse anti–myelin basic protein (MBP; 1:1,000; myelin marker, Abcam; Cat# ab62631 Lot# RRID:AB_956157); mouse anti–beta-amyloid precursor protein (β-APP; 1:500; amyloid peptide marker, Abcam; Cat# ab11132 Lot# RRID:AB_297770); rabbit anti-Nogo-A (1:500; myelin-derived neurite growth inhibitor, Abcam; Cat# ab62024 Lot# RRID:AB_956171); mouse anti–post-synaptic density 95 (PSD-95; 1:500; post-synaptic density protein, Merck Millipore, Burlington, MA; Cat# MAB1596 Lot# RRID:AB_2092365); and rabbit anti-synaptophysin (1:1000; presynaptic protein, Cell Signaling Technology; Cat# 5461S Lot# RRID:AB_10698743), all diluted in 5% bovine serum albumin solution, and membranes were incubated overnight at 4°C. The primary antibody was removed and the blots were washed in Tris-buffered saline (TBS)/Tween and incubated (1 h, room temperature) in horseradish-peroxidase–conjugated secondary antibodies (1:10,000; Jackson ImmunoResearch Labs, West Grove, PA; Cat# 323-005-021 Lot# RRID:AB_2314648). Reactive proteins were visualized using enhanced chemiluminescence (VWR International, Franklin, MA). Optical density was determined using ImageJ software (NIH, RRID:SCR_003070). All membranes were also incubated with a mouse polyclonal antibody for β-actin (1:4,000; Sigma-Aldrich; Cat# A5316 Lot# RRID:AB_476743). Protein level was expressed as relative optical density, that is, the optical density of the band revealed by the primary antibody, divided by the optical density of β-actin in the same lane.

### Phospholipid fatty acid analysis

PL fatty acid content of plasma was determined as described.^48^ Lipids were extracted using the method of Folch and colleagues,^49^ with 0.01% (w/v) 2,6-di-tert-butyl-p-cresol (butylated hydroxytoluene) added as antioxidant. Total PLs were isolated by thin-layer chromatography, and the PL fatty acid composition was measured after transesterification with 14% boron trifluoride in methanol. Fatty acids were identified by gas chromatography with a flame ionisation detector (Agilent 7820A; Agilent Technologies, Santa Clara, CA) using an Omegawax^®^ capillary column (15 m × 0.10 mm × 0.10 μm). Identity was confirmed by retention times compared to standards, and quantification was performed on peak areas with ChemStation software (Agilent Technologies). Corrections were made for variations in detector response, and values of fatty acids were normalized to 100% and expressed as wt %.

PL content of the cerebellum was measured as previously described.^50^ Neutral and acidic PLs were isolated from a total lipid extract by solid-phase extraction using Isolute^®^ bonded phase aminopropyl columns (Kinesis Health Technologies, Dublin, UK). The neutral PL extract was separated into PC and PE by thin-layer chromatography and the phosphate content measured. The results were normalized to 100-mg wet tissue weight.

### Statistical analysis

Statistical analyses were done out using GraphPad Prism software (version 5; RRID:SCR_002798; GraphPad Software Inc., La Jolla, CA). Data are expressed as mean ± standard error of the mean (SEM). Groups were compared using one- or two-way analysis of variance (ANOVA), followed by different post-hoc tests for multiple comparisons, and significance level set at *p* < 0.05.

## Results

### Fortasyn Connect supplementation improves sensorimotor impairment after traumatic brain injury

TBI survivors experience sensorimotor impairment, including paresis, postural imbalance, gait disturbance,^51^ and disrupted startle response.^52^ TBI leads to bradykinesia, abnormal sway, and impaired reaction time.^53,54^ Early balance impairment is a predictor of worse outcome post-TBI. Sensorimotor problems improve over time, although some deficits may persist beyond the first 1–2 years post-trauma.^51^

**FIG. 2.**
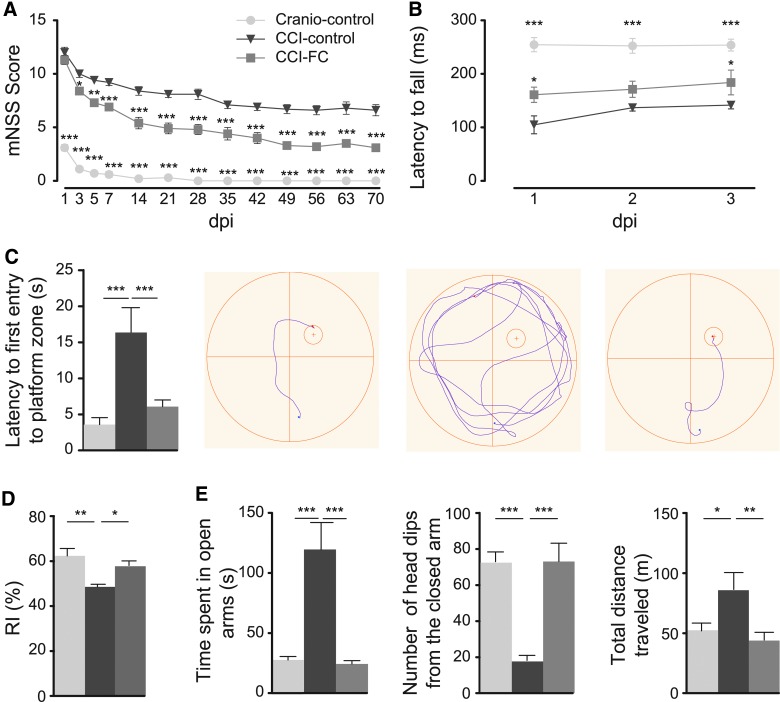
Integrated neurological function and motor assessments. mNSS (**A**) and Rotarod (**B**). (A) From 5 dpi and thereafter, CCI-FC animals showed a significant improvement compared to the CCI-control group (two-way ANOVA, *p* < 0.0001; *F*_(2,27)_ = 320.8; Bonferroni's post-hoc test, **p < 0.01; ***p < 0.001); the craniotomy-control group showed significant improvement from 1 dpi and nearly no deficits after 14 dpi (***p < .001 compared to CCI-control). (B) On 1 and 3 dpi, CCI-FC and craniotomy-control mice showed marked improvement compared to CCI-control mice (one-way ANOVA, *p* < 0.0001; *F*_(2,26)_ = 42.31; Bonferroni's post-hoc test, *p < 0.05; ***p < 0.001, respectively). Data are means ± SEM of 10 animals/group. Cognitive performance assessments. MWM (**C**) and NOR (**D**). (C) A significant reduction in latency to the first entry to the platform-quadrant was observed in CCI-FC and craniotomy control animals in the probe test (one-way ANOVA, *p* = 0.0005; *F*_(2,27)_ = 10.17; Bonferroni's post-hoc test, **p < 0.01; ***p < 0.001, respectively) compared to CCI-control animals. Underneath, an illustration of the track of an animal, from release into the water until it first entered the platform quadrant. (D) A significant increase in the time spent exploring a novel object, compared to the familiar one, was observed in both CCI-FC and craniotomy control mice compared to CCI-control mice (one-way ANOVA, *p* < 0.003; *F*_(3,36)_ = 5.596; Bonferroni's post-hoc test, *p < 0.05; **p < 0.01, respectively). Results expressed as the recognition index (RI) %: the time spent investigating the novel object relative to the total time of object investigation. Data are means ± SEM of 10 animals per group. Anxiety assessment. Elevated zero maze (EZM; **E**). CCI-FC and craniotomy-control mice showed limited exploration of the unfamiliar environment, shown by i) reduced preference for open zones, as reflected in total time spent in the open zone (one-way ANOVA, *p* = 0.0004; *F*_(2,12)_ = 16.52; Bonferroni's post-hoc test, ***p < 0.001) compared to CCI-control animals; ii) a lower number of head dips (one-way ANOVA, *p* = 0.0001; *F*_(2,12)_ = 21.92; Bonferroni's post-hoc test, ***p < 0.001); and iii) a reduced total distance traveled during the 5-min trial (one-way ANOVA, *p* = 0.0068; *F*_(2,12)_ = 7.799; Bonferroni's post-hoc test, **p < 0.01; *p < 0.05, respectively). Data are means ± SEM of 10 animals/group. ANOVA, analysis of variance; CCI, controlled cortical impact; dpi, days post-injury; FC, Fortasyn^®^ Connect; MWM, Morris water maze; mNSS, Modified Neurological Severity Score; NOR, novel object recognition; SEM, standard error of the mean.

Sensorimotor impairment was assessed with mNSS, Rotarod, and gait analysis. All groups showed a decrease in TBI-induced impairment over 70 days, but a significant improvement was observed in CCI FC-treated animals as early as the third day post-trauma, compared to the CCI-control diet group. This difference in mNSS was maintained until the end (two-way ANOVA, *p* < 0.0001; *F*_(2,27)_ = 320.8; Fig. 2A). Craniotomy control animals showed only a transient impairment. Tasks where impairment was the most prolonged were the tasks related to balance, that is, the round and triangular stick balance tasks and the 1-cm beam walk task.

Rotarod results in the 3 days post-injury revealed better performance in FC-treated animals (one-way ANOVA, *p* < 0.0001; *F*_(2,26)_ = 42.31; Fig. 2B). Latency to fall off the Rotarod was significantly higher in FC-supplemented animals, compared to control CCI animals. The craniotomy-control group showed minimal coordination and balance impairment.

Gait analysis at 2 dpi using the CatWalk system revealed impaired locomotion in CCI-control diet animals. A long print length reflects foot dragging and less control of the foot. We also noticed after injury a smaller print width and a smaller print area within the left hindpaw (LH) in the control diet group, which indicated a poor control of the paw placement. The FC-treated group showed a decrease in print length for the LH compared to the control diet group. We also assessed the stand index (SI), which measures the speed at which the paw loses contact with the glass plate. The SI was higher in CCI-control diet animals compared to the other groups, emphasizing a poor support of the LH during locomotion. The step cycle, which runs from the initial contact to the following contact of the same paw, was longer in the CCI-control diet group. We also noticed a slower body speed in this group compared to CCI-FC and craniotomy-control animals. These latter two parameters may reflect a motor “clumsiness” that is a consequence of the impaired relationship between the placement of paws within a step. The measurements indicated that TBI reduced interlimb coordination and the FC diet led to partial improvement (Supplementary Fig. 3) (see online supplementary material at http://www.liebertpub.com).

### Fortasyn Connect FC improves spatial memory deficits in the Morris water maze and reduces novel object recognition impairment after traumatic brain injury

TBI leads to deficits in cognition (e.g., memory, attention, and information-processing speed), which are TBI-severity dependent. The temporal lobes are vulnerable in TBI, in part because of their location in the skull. The hippocampus plays a key role in memory processing, recognition, acquisition, and storage of the contextual details and temporal order of stimuli; hippocampal atrophy is related to injury severity.^55,56^ Spatial memory was assessed using the MWM, a test used to detect impairments in hippocampal-dependent learning and memory.^57^ CCI led to a disruption of the task acquisition, and FC-supplemented animals did not show significant differences compared to injured animals fed the control diet (Supplementary Fig. 4) (see online supplementary material at http://www.liebertpub.com). The probe trial revealed a major impairment post-CCI (one-way ANOVA, *p* = 0.0005; *F*_(2,27)_ = 10.17; Fig. 2C), which was reversed by the FC-supplemented diet.

The NOR task is another widely used model for the investigation of memory. Observations in primates and rodents have shown the importance of the parahippocampal regions of the temporal lobe (perirhinal, entorhinal, and inferior temporal cortices) for visual object recognition memory.^58^

In the NOR, craniotomy-control mice spent the highest proportion of time with the novel object (one-way ANOVA, *p* = 0.003; *F*_(3,36)_ = 5.596; Fig. 2D). CCI resulted in a significant reduction in the RI, and this reduction was partly reversed by FC.

### Fortasyn Connect reverses the disrupted behaviour in the elevated zero maze after traumatic brain injury

TBI survivors frequently present with a delayed emergence of increased anxiety, agitation and disinhibition, and aggressive behavior.^59^ These changes in personality disrupt relationships and hinder rehabilitation. Anxiolytic and anxiogenic factors can be tested using the EPM, during which animals display a preference for dark spaces. In a CCI study, it was shown that at 21 days post-injury, mice display a reduced anxiety.^60^

We tested the impact of CCI in the EZM, a modification of the EPM that eliminates the central region of the maze, thus focusing the analysis on the behavior in the closed and open spaces.^61^ CCI-injured mice fed with control diet showed a reduced level of anxiety compared to control-craniotomy mice, resulting in an increased exploration time in the open areas. This correlated with a lower number of head dips from the closed arms and a higher distance traveled compared to CCI-FC and craniotomy-control mice. Therefore, TBI led to a disinhibition of mice, and in CCI mice receiving FC, the multi-nutrient reduced this behavioral disinhibition (one-way ANOVA; total distance, *p* = 0.0068, *F*_(2,12)_ = 7.799; head dips, *p* = 0.0001, *F*_(2,12)_ = 21.92; time spent in open arms, *p* = 0.0004, *F*_(2,12)_ = 16.52; Fig. 2E).

### Fortasyn Connect–supplemented animals show a significant reduction in lesion size

TBI is associated with a reduction in brain volume, which continues for years after the injury.^62^

**FIG. 3.**
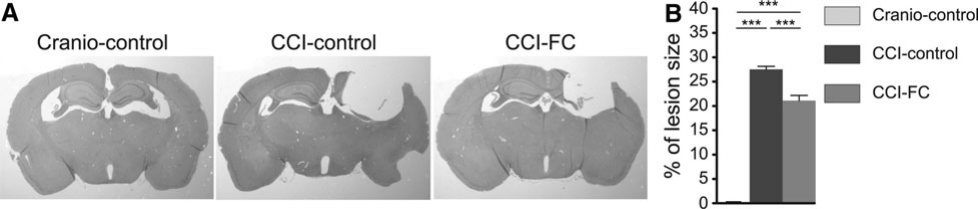
Lesion size (**A** and **B**). (A) Sections stained with H&E showing differences in lesion size. (B) Graph showing a significant reduction in lesion size in CCI-FC mice compared to CCI-control versus craniotomy-control mice, at 70 days post-TBI (one-way ANOVA, *p* < 0.0001; *F*_(2,33)_ = 382.5; Bonferroni's post-hoc test, ***p < 0.001). Data are means ± SEM of 5 animals/group. Data are means ± SEM of 5 animals/group. ANOVA, analysis of variance; CCI, controlled cortical impact; dpi, days post-injury; FC, Fortasyn^®^ Connect; H&E, hematoxylin and eosin; SEM, standard error of the mean; TBI, traumatic brain injury.

Analysis of lesion size showed that, at 70 dpi, there was a significant loss of tissue, with almost total loss of ipsilateral hippocampus. FC supplementation significantly decreased lesion size (one-way ANOVA; *p* < 0.0001, *F*_(2,33)_ = 382.5; Fig. 3A,B). There was no difference in neuronal numbers (neuronal nuclei–positive cells) perilesionally, between the three groups (not shown).

### Fortasyn Connect supplementation leads to a decrease in microglia activation and alters the astrocyte response post-injury

**FIG. 4.**
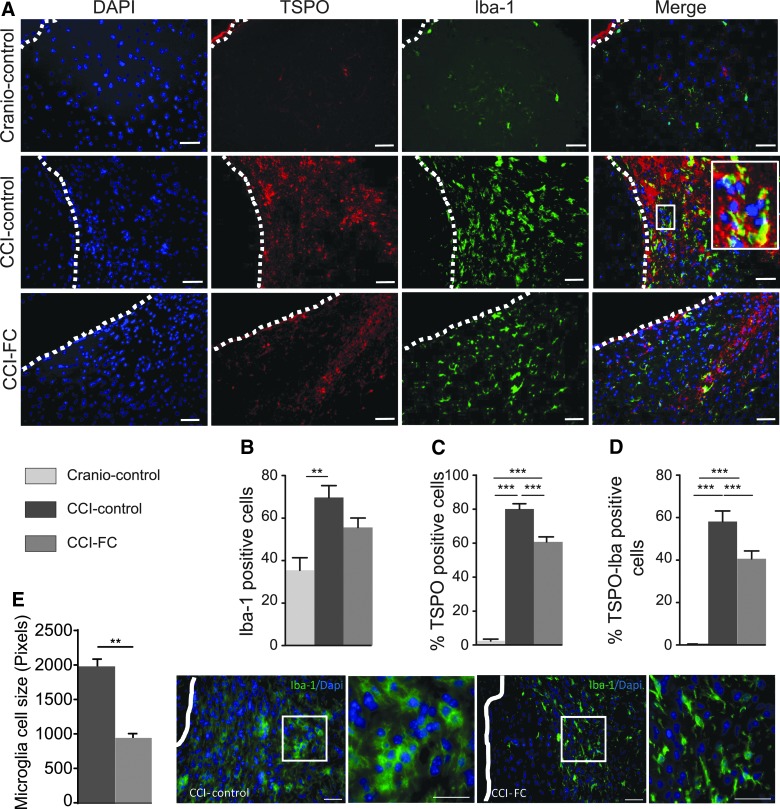
Neuroinflammatory response. (**A**) Images of DAPI, TSPO, Iba-1, and double-labeled TSPO/Iba-1 cells. Note the different microglia morphology (activation): amoeboid versus ramified. Scale bars = 100 μm. To show colocalization, we enlarged the area marked with a rectangle. Scale bars = 100 μm. Immunohistochemistry quantification, around the lesion border, of (**B**) %Iba-1–positive cells (one-way ANOVA, *p* = 0.0032; *F*_(2,11)_ = 10.1; Bonferroni's post-hoc test, **p < 0.01), (**C**) %TSPO-positive cells (one-way ANOVA, *p* < 0.0001; *F*_(2,11)_ = 273.7; Bonferroni's post-hoc test, ***p < 0.001), and (**D**) colocalized TSPO- and Iba-1–positive cells (one-way ANOVA, *p* < 0.0001; *F*_(2,11)_ = 77.97; Bonferroni's post-hoc test, ***p < 0.001). (**E**) Microglia cell-size analysis (Mann-Whitney U test, **p = 0.0079) and corresponding images, showing the differences in cell size. Insets show the clear morphological differences. Scale bars = 100 μm. Data are means ± SEM of 5 animals/group. ANOVA, analysis of variance; CCI, controlled cortical impact; DAPI, 4’,6-diamidino-2-phenylindole; FC, Fortasyn^®^ Connect; Iba-1, ionized calcium binding adaptor molecule 1; SEM, standard error of the mean; TSPO, translocator protein.

Microglia are brain immune cells derived from the yolk sac,^63^ with phagocytic and antigen-presenting properties,^64^ which exert constant brain surveillance.^65^ Their activation is a hallmark of TBI^66^ and is reflected in morphological changes, from a quiescent ramified morphology^67^ to an amoeboid aspect.^68^ TBI leads to microglial activation, which can be detected in humans years after injury.^69^ This long-lasting response has also been described in mouse CCI.^38^ One of the markers of microglia activated post-TBI is the TSPO, located in the mitochondrial membrane.^70^ Positron emission tomography imaging has shown an increase in TSPO post-TBI in humans^71^ and in rat CCI.^72^ We investigated the microglial response with the Iba-1 and TSPO markers. The percentage of Iba-1–positive cells around the lesion was higher in the CCI-control diet group compared to FC-CCI and craniotomy groups (one-way ANOVA, *p* = 0.0032; *F*_(2,11)_ = 10.1; Fig. 4A,B). FC led to an overall decrease in TSPO-positive cells compared to control diet–treated CCI animals (one-way ANOVA, *p* < 0.0001; *F*_(2,11)_ = 273.7; Fig. 4A,C). Staining for TSPO and Iba-1 showed a significantly lower number of double-stained cells in the FC-supplemented group compared to the CCI group on control diet (one-way ANOVA, *p* < 0.0001; *F*_(2,11)_ = 77.97; Fig. 4A,D). Notable differences in glia morphology were observed between the CCI-control diet group (predominantly amoeboid), CCI-FC diet group (less amoeboid), and craniotomy-control group (predominantly ramified). Therefore, we conducted an additional cell-size analysis to underline morphological differences in the two CCI groups. Microglial cell size in the CCI-control diet group was significantly larger compared to the CCI-FC group (Mann-Whitney U test, *p* = 0.0079; Fig. 4E).

Astrocytes are part of the brain macroglia and are activated post-TBI^73,74^; this is attributed to the traumatic impact *per se*, as well as the ischaemia, disruption of the blood–brain barrier, inflammation, and other metabolic changes post-injury. The astrogliotic reaction is reflected in both proliferation and cell hypertrophy.^75,76^ These processes can be followed using quantification of intermediate filament proteins and by labeling the newly formed astrocytes. *In vivo* imaging shows the heterogeneity of the astrocytic response post-injury in mice, that is, the existence of proliferative and nonproliferative subpopulations.^75^ Post-injury, astrocytes can exert both damaging and protective effects. Some astrocytes are lost in the first days post-injury,^77^ whereas later on these cells are involved in the formation of the perilesion scar.^78^ Ablation of proliferative astrocytes post-CCI in mice leads to a worse outcome, suggesting that the astrocytic response and scar limit the injury impact.^79^ During remodeling post-TBI, astrocytes may help maintain neuronal excitability.^80^

**FIG. 5.**
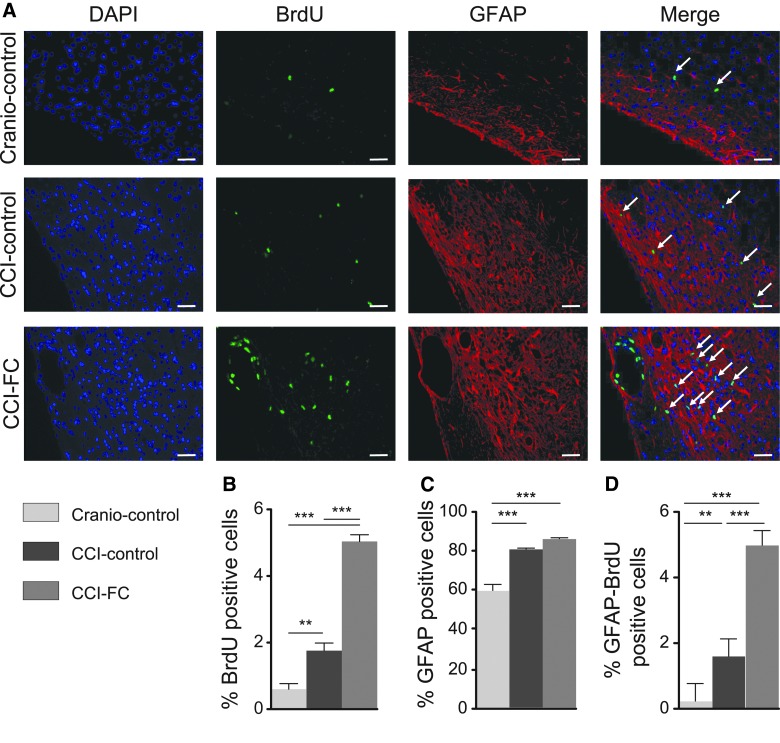
Astrocyte response post-injury. (**A**) Images of DAPI, BrdU, GFAP, and double-labeled GFAP/BrdU cells. Arrows show colocalization. Scale bars = 100 μm. Immunohistochemistry quantification, around the lesion border, of (**B**) %BrdU-positive cells (one-way ANOVA, *p* < 0.0001; *F*_(2,12)_ = 131.5; Bonferroni's post-hoc test, **p < 0.01; ***p < 0.001). (**C**) %GFAP-positive cells (one-way ANOVA, *p* < 0.0001; *F*_(2,12)_ = 40.37; Bonferroni's post-hoc test, ***p < 0.0001) and (**D**) colocalised %GFAP- and BrdU-positive cells (one-way ANOVA, *p* < 0.0001; *F*_(2,12)_ = 119.4; Bonferroni's post-hoc test, ***p < 0.0001). Data are means ± SEM of 5 animals per group. ANOVA, analysis of variance; BrdU, bromodeoxyuridine; CCI, controlled cortical impact; DAPI, 4’,6-diamidino-2-phenylindole; FC, Fortasyn^®^ Connect; GFAP, glial fibrillary acidic protein; SEM, standard error of the mean.

CCI led to an increase in astrocyte staining in the perilesional area versus craniotomy-only animals, without any differences between the control and FC-supplemented diet (one-way ANOVA, *p* < 0.0001; *F*_(2,12)_ = 40.37; Fig. 5A,C). Determination of the newly formed astrocytes, that is, cells double-labeled with BrdU and GFAP, showed an intense proliferation at 70 dpi, which was amplified by FC supplementation (one-way ANOVA, *p* < 0.0001; *F*_(2,12)_ = 119.4; Fig. 5A,D).

### Fortasyn Connect supplementation modulates cell proliferation and neurogenesis after controlled cortical impact

**FIG. 6.**
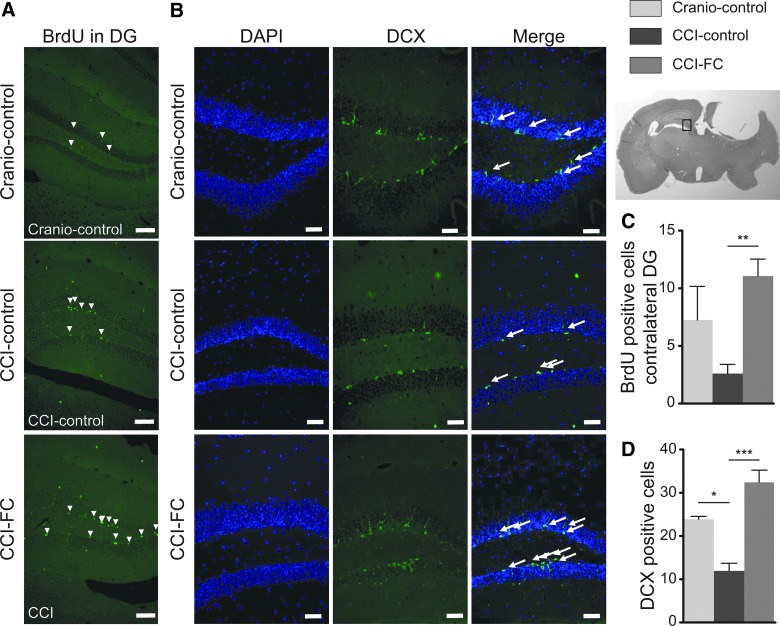
Cell proliferation and neurogenesis. (**A**) Images of BrdU in the contralateral dentate gyrus (DG). Scale bars = 25μm. (**C**) Immunohistochemistry quantification of BrdU-positive cells in the contralateral DG (one-way ANOVA, *p* = 0.0098; *F*_(2,9)_ = 8.069; Bonferroni's post-hoc test, **p < 0.01). Data are means ± SEM of 5 animals/group. Number of positive doublecortin (DCX) cells in the contralateral DG. (**B**) Images of DCX in the contralateral DG. Scale bars = 25μm. (**D**) Quantification of DCX-positive cells in the contralateral DG (one-way ANOVA, *p* = 0.0009; *F*_(2,6)_ = 27.64; Bonferroni's post-hoc test, *p < 0.05; ***p < 0.001). Data are means ± SEM of 5 animals/group. ANOVA, analysis of variance; BrdU, bromodeoxyuridine; CCI, controlled cortical impact; DAPI, 4’,6-diamidino-2-phenylindole; FC, Fortasyn^®^ Connect; SEM, standard error of the mean.

CCI leads to a robust increase in cell proliferation, which involves both glial progenitors and cells in the neurogenic niche.^81^ The increase in the first hours post-injury is in the subventricular zone, whereas later on an increase in BrdU-labeled cells can be detected throughout the lesioned hemisphere, and also contralaterally.^82^ TBI is also associated with changes in neurogenesis. A decreased number of immature neurons has been reported in mouse CCI in the first week post-injury, ipsilaterally and contralaterally.^83^ The assessment of the proliferating cells post-CCI showed a significantly higher percentage of BrdU-positive cells perilesionally compared to the craniotomy control group. Treatment with FC led to a markedly increased cell proliferation perilesionally (one-way ANOVA, *p* < 0.0001; *F*_(2,12)_ = 131.5; Fig. 5A,B), and contralaterally, in the dentate gyrus (DG; one-way ANOVA, *p* = 0.0098; *F*_(2,9)_ = 8.069; Fig. 6A,C). In the contralateral hippocampus, CCI decreased the number of immature neurons (DCX-positive cells), to less than half the value detected in craniotomy-control animals (one-way ANOVA, *p* = 0.0009; *F*_(2,6)_ = 27.64; Fig. 6B,D). Supplementation with FC restored the number of immature neurons to the level observed in craniotomy-control animals.

### Fortasyn Connect supplementation leads to protection of myelin and oligodendrocytes after traumatic brain injury

Imaging and post-mortem studies show that altered white mater integrity is a major consequence of TBI.^84–86^ Oligodendrocytes have a key role in the white matter repair post-trauma.^87^ The demyelination response post-injury in mice is already detected 3 days post-injury, with evidence of remyelination by the end of the first week post-injury.^88^

**FIG. 7.**
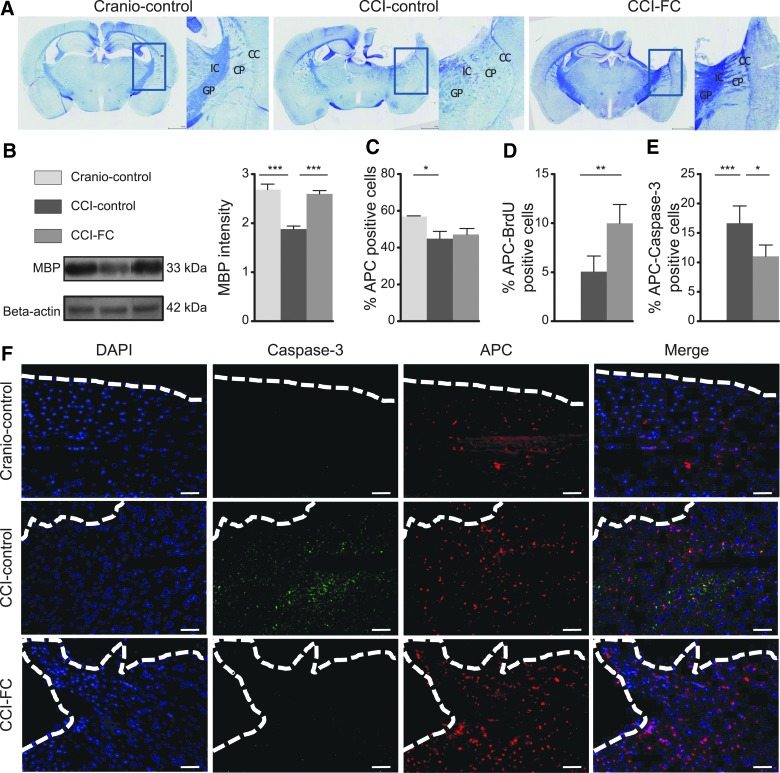
Myelin. (**A**) Coronal brain sections stained with Luxol fast blue (LFB). Note differences in the internal capsule (IC), globus pallidus (GP)-external segment, caudate-putamen (CP), and corpus callosum (CC) regions (areas marked with rectangles and enlarged). In the CCI-FC and craniotomy control groups, myelin-stained tracts look continuous, in contrast with a dotted pattern seen in the CCI-control group, both ipsilateral and contralateral to the injury site. (**B**) Myelin basic protein (MBP) levels by western blot. MBP 20kDa was significantly increased in the CCI-FC and craniotomy-control groups (one-way ANOVA, *p* < 0.0001; *F*_(2,12)_ = 27.81; Bonferroni's post-hoc test, ***p < 0.001) compared to CCI-control mice. Oligodendrocytes. (**C**) Quantification of oligodendrocytes (%APC-positive cells; one-way ANOVA, *p* = 0.0417; *F*_(2,9)_ = 4.619; Bonferroni's post-hoc test, *p < 0.05). (**D**) Dual-staining APC and BrdU (one-way ANOVA, *p* = 0.0028; *F*_(2,9)_ = 12.09; Bonferroni's post-hoc test, **P < 0.01). (**E**) Dual-staining APC with caspase-3 (one-way ANOVA, *p* = 0.0009; *F*_(2,9)_ = 16.9; Bonferroni's post-hoc test, *p < 0.05; ***p < 0.001) and (**F**) images of oligodendrocytes, caspase-3, and DAPI around the lesion border. Scale bar = 100 μm. Data are means ± SEM of 5 animals/group. ANOVA, analysis of variance; APC, adenomatous polyposis coli; BrdU, bromodeoxyuridine; CCI, controlled cortical impact; DAPI, 4’,6-diamidino-2-phenylindole; FC, Fortasyn^®^ Connect; SEM, standard error of the mean.

Luxol fast blue staining showed myelin disruption post-CCI, particularly in the caudate-putamen and internal capsule. Qualitative analysis indicated preserved patterns of myelin in CCI-FC and craniotomy-control mice (Fig. 7A), whereas CCI-control diet animals showed severe disruption. MBP showed a statistically significant decrease in CCI mice fed with the control diet, with the levels fully restored after FC supplementation (one-way ANOVA, *p* < 0.0001; *F*_(2,12)_ = 27.81; Fig. 7B). Injury reduced oligodendrocytes perilesionally. This CCI-induced reduction was not modified by the FC diet (one-way ANOVA, *p* = 0.00417; *F*_(2,9)_ = 4.619; Fig. 7C). However, dual staining with APC and BrdU showed a much higher percentage of double-stained cells in the CCI-FC diet group compared to the other groups (one-way ANOVA, *p* = 0.0028; *F*_(2,9)_ = 12.09; Fig. 7D). Further, double staining of APC with caspase-3 showed a significantly lower percentage of cells in the FC diet-CCI group compared to the CCI-control diet group (one-way ANOVA, *p* = 0.0009; *F*_(2,9)_ = 16.9; Fig. 7E), suggesting an ongoing higher rate of apoptosis in the untreated injured group. Overall, these findings support a protective effect of FC on white matter and a significant support of the proliferative pool of oligodendrocytes.

### Fortasyn Connect modulates changes in synaptic markers after controlled cortical impact/synaptophysin and post-synaptic density 95

It has been shown that even mild experimental TBI, which is not associated with development of a lesion cavity, leads to dendritic degeneration of the apparently spared neurons in the perilesional area, and to loss of synapses, as evidenced using synaptophysin immunostaining,^89^ as early as 3 days post-injury. Further, in the same mild CCI mouse model, altered dendritic spine density and marked dendritic beading and swelling were reported in the hippocampus.^13^ More recently, it was shown that, 24 h post-injury, dendritic spine loss could be detected both in the ipsi- and contralateral hemisphere.^90^ Alterations are persistent, given that changes in the cortical dendritic arbor have been reported at 4 months post-CCI.^91^ A significant decrease in the post-synaptic marker, PSD-95, has been reported post-CCI, at a time when animals displayed cognitive impairment.^92^

**FIG. 8.**
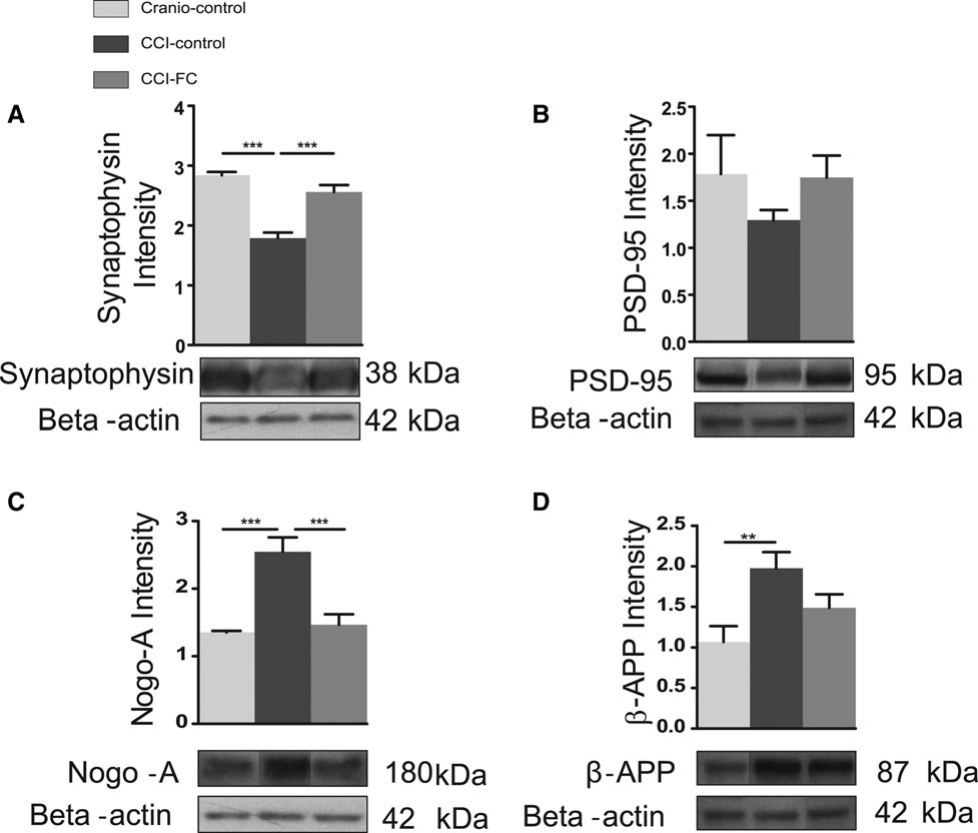
Synaptic markers. Graph and cropped gels of western blot analysis of protein levels of (**A**) synaptophysin; 38 kDa (one-way ANOVA, *p* < 0.0001; *F*_(2,12)_ = 35.5; Bonferroni's post-hoc test, ***p < 0.001) and (**B**) PSD-95; 95kDa (one-way ANOVA, *p* = 0.4188; *F*_(2,12)_ = 0.9365; Bonferroni's post-hoc test, ^#^*p* < 0.06) were analyzed by western blot. Data are means ± SEM of 5 animals/group. Neurite outgrowth inhibitor and amyloid load. (**C**) Graph and cropped gels of western blot analysis of protein levels of Nogo-A; 180 kD (one-way ANOVA, *p* = 0.0002; *F*_(2,12)_ = 18.68; Bonferroni's post-hoc test, ***p < 0.001) and (**D**) β-APP; 87 kDa (one-way ANOVA, *p* = 0.0163; *F*_(2,12)_ = 5.915; Bonferroni's post-hoc test, **p < 0.01) were analyzed in both the CCI-FC and craniotomy control mice compared to CCI-control mice. Data are mean ± SEM of 5 animals/group. β-actin was used as loading control. ANOVA, analysis of variance; β-APP, beta-amyloid precursor protein; CCI, controlled cortical impact; FC, Fortasyn^®^ Connect; PSD-95, post-synaptic density 95; SEM, standard error of the mean.

The CCI group on a control diet showed a significant decrease in synaptophysin. In contrast, in the CCI-FC group, the level of synaptophysin was almost the same as that found in the craniotomy-control group (one-way ANOVA, *p* < 0.0001; *F*_(2,12)_ = 35.5; Fig. 8A). We found a similar trend in the post-synaptic protein, PSD-95, which was reduced post-CCI and restored after FC supplementation—however, these changes were not statistically significant (one-way ANOVA, *p* = 0.4188; *F*_(2,12)_ = 0.9365; Fig. 8B).

### Fortasyn Connect decreases the neurite outgrowth inhibitor and amyloid load

TBI leads to compensatory neuroplasticity, and a major impediment to recovery is the limited axonal regeneration. Factors such as the myelin-associated inhibitor, Nogo-A, are linked to limited plasticity post-injury. An increase in Nogo-A was observed within the first week post-injury, after rat fluid percussion injury and mouse CCI.^93,94^

The level of the axon growth inhibitor, Nogo-A, was upregulated by injury and significantly reduced by the FC diet (one-way ANOVA, *p* = 0.0002; *F*_(2,12)_ = 18.68; Fig. 8C).

One of the burdens associated with TBI is the increased incidence of AD in TBI survivors.^95^ Changes in β-APP post-TBI have been reported at both early and delayed times post-injury. An increase in β-APP has been found post-CCI in rats, up to 3 days after injury.^96^ We found that the tissue level of β-APP in the lesioned area was significantly increased, and reduced by the FC diet (one-way ANOVA, *p* = 0.0163; *F*_(2,12)_ = 5.915; Fig. 8D).

### Fortasyn Connect alters plasma phospholipid levels of arachidonic acid, eicosapentaenoic acid, and docosahexaenoic acid

**FIG. 9.**
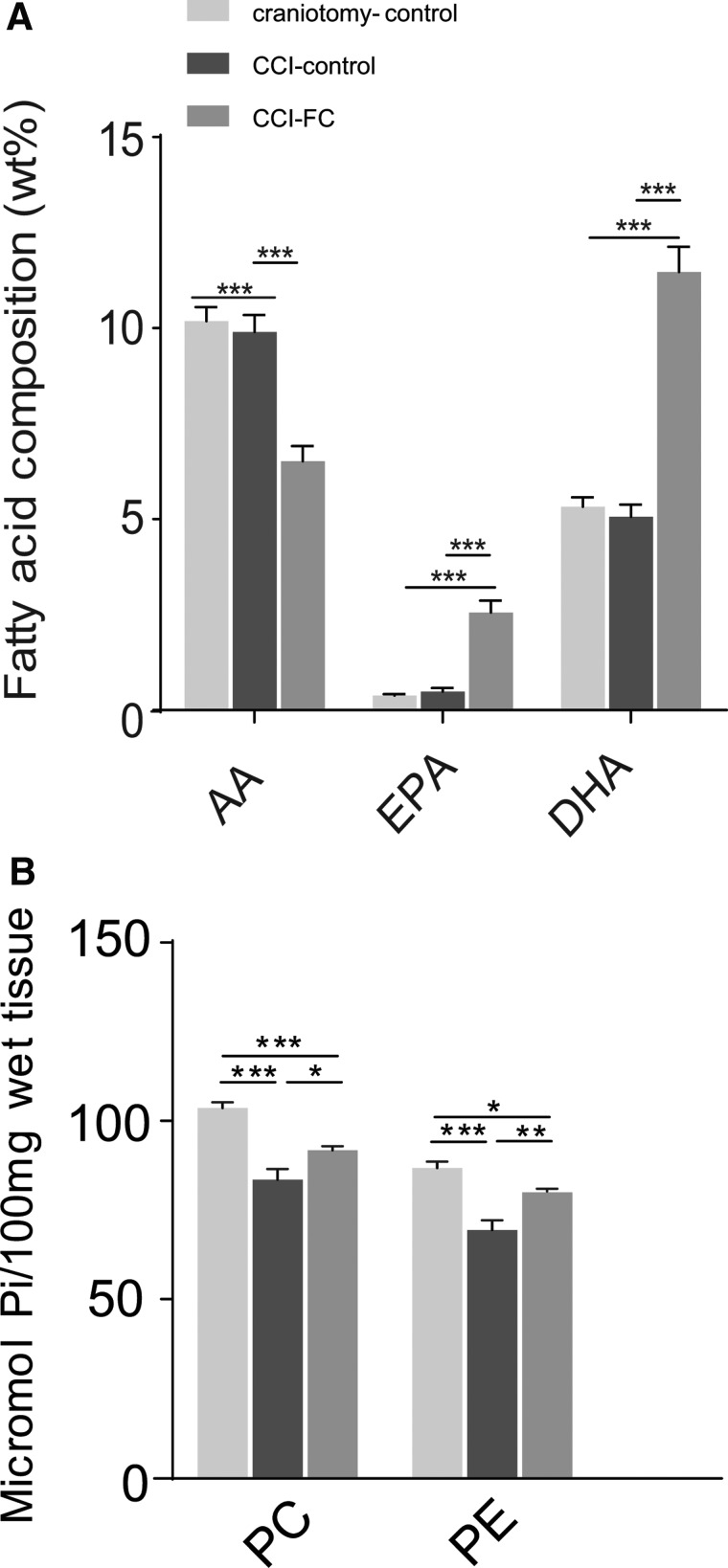
Phospholipid fatty acid composition in plasma and tissue phospholipids. In plasma (**A**), a significant reduction in AA and increases in EPA and DHA in CCI-FC mice compared to CCI-control and craniotomy-control at 70 days post-TBI (one-way ANOVA, *p* < 0.0001; *F*_(2,93)_ = 20.45; Bonferroni's post-hoc test, ***p < 0.001). Data are means ± SEM of 5 animals/group. In cerebellum (**B**), a reduction in tissue PC levels by 20% in injured animals on the control diet versus craniotomy controls, reduced to 11% in the FC supplementation group compared to craniotomy controls (one-way ANOVA, *p* < 0.0001; *F*_(2, 3)_ = 26.99; Bonferroni's post-hoc test, ***p < 0.001). Data are means ± SEM of 5 animals/group. PE levels decreased by 21% in the injured animals on the control diet versus craniotomy-only, whereas after FC supplementation the difference versus craniotomy controls was only 8% (one-way ANOVA, *p* < 0.0001; *F*_(2, 3)_ = 20.82; Bonferroni's post-hoc test, ***p < 0.001). Data are means ± SEM of 5 animals/group. AA, arachidonic acid; ANOVA, analysis of variance; CCI, controlled cortical impact; DHA, docosahexaenoic acid; EPA, eicosapentaenoic acid; FC, Fortasyn^®^ Connect; PC, phosphatidylcholine; PE, phosphatidylethanolamine; SEM, standard error of the mean; TBI, traumatic brain injury.

Fatty acid composition of plasma PL at 70 dpi showed that the FC-supplemented group had a significantly higher level of EPA and DHA, compared to the CCI-control diet and craniotomy-control. In contrast, a significantly lower level of AA was found in the CCI-FC group compared to the other two groups (two-way ANOVA, *p* < 0.0001; *F*_(2,93)_ = 20.45; Fig. 9a), therefore resulting in a higher DHA/AA ratio. No changes were observed in the other fatty acids (Supplementary Fig. 5) (see online supplementary material at http://www.liebertpub.com). The ratio DHA/AA was reported to be lower at 3 months post-CCI,^21^ in particular in the PE fraction of plasma PL; therefore, our results show that the FC-based intervention could help improve the DHA/AA ratio post-injury.

### Fortasyn Connect alters the tissue levels of phosphatidylcholine and phosphatidylethanolamine

Analysis of PL composition in the cerebellum showed that PC levels decreased versus craniotomy controls by 20% post-CCI, and after FC supplementation the difference versus craniotomy controls was reduced to 11%. PE levels decreased by 21% in the injured animals on the control diet versus craniotomy-only, whereas after FC supplementation the difference was only 8% (two-way ANOVA, *p* < 0.0001; *F*_(1,26)_ = 47.12; Fig. 9B).

## Discussion

In this study, we show, for the first time, that a specialized medical multi-nutrient, which provides PL precursors and which has recently been shown to reduce hippocampal atrophy in prodromal AD,^97^ induces a significant neurological improvement and reduces the impact of injury when administered post-TBI. The intervention with FC in a murine injury model markedly improved outcome and reduced tissue loss.

FC improved gait, balance and motor coordination, spatial and recognition memory, and it corrected behavioral disinhibition. The wide range of improvements throughout the period post-injury were observed in parallel with beneficial effects on tissue.

Focal contusion TBI, such as that induced in CCI, ultimately creates cavitation at the injury epicentre. The tissue loss is linked to the sensorimotor and cognitive deficits.^98^ The lesion was decreased after PL precursor supplementation, and lesion reduction could be correlated with improved outcome.^99^ The multi-nutrient also modified neuroinflammation. Microglia play a major role in the inflammatory response post-TBI.^100^ FC reduced the activated microglia perilesionally. Activated microglia express the cholesterol transporter protein, TSPO, and CCI led to an increase in TSPO. Other studies have reported an increase in TSPO in models of TBI^72,101^ and TBI patients.^71^ FC supplementation significantly reduced TSPO expression at 70 days post-CCI.

Microglial activation may relate to white matter disruption.^102^ Colocalization of MBP immunoreactivity with microglia post-TBI suggests that myelin fragments could provide a persistent trigger for inflammation, by stimulating microglial activation.^103^ The increased activated microglia post-TBI also correlate with elevated tissue levels of β-amyloid (Aβ).^104^ The reduction in activated microglia around the lesion, after FC, may have contributed to axonal protection by lessening the accumulation of Aβ. This accumulation occurs in damaged axons post-TBI, and axonal injury can be detected using labeling for β-APP.^16,74,105,106^ Our data show an increased expression of β-APP in injured tissue, as reported previously,^107,108^ and supplementation with FC partially reversed this.

Astrocytes have a pivotal role post-TBI and undergo reactive astrogliosis.^80^ Astrocytes control leukocyte infiltration, blood–brain barrier repair, and neuronal degenerative processes post-TBI and act as buffers for neurotransmitter excess and modulate neurovascular coupling.^109^ Disruption of the links between astrocytes and oligodendrocytes can result in demyelination, in parallel with motor impairment.^110^ Astrocytes have a role in synaptic plasticity and neural circuit reorganization, and remyelination. We show post-CCI an increase in newly born astrocytes post-injury and this to a greater extent in the FC group. This may contribute to tissue repair/regeneration processes.

We also show that the PL precursor combination protects white matter and oligodendrocytes post-TBI. We found restored levels of MBP, a decreased level of oligodendrocyte death, and more newly formed oligodendrocytes. There is a strong relationship between axon and myelin integrity—the proliferation, differentiation, and maintenance of oligodendrocytes require axon-derived signals and there is a continuous cross-talk and mutual dependence.^111^

FC stimulated neurogenesis, and treatments that stimulate neurogenesis, especially in the late phase post-injury, could contribute to circuit restoration.^112–115^ Functional recovery also depends on axonal regeneration and sprouting, which underlie the neuroplastic changes that accompany recovery.^116^ We found that the sustained supply of FC significantly reduced Nogo-A, a myelin component that inhibits axonal growth,^117^ thus showing that FC enables a supportive environment for axons. These results are in agreement with Wurtman and colleagues, who showed lower levels of Nogo-A in aged rats after supplementation with these precursors,^118^ and in accord with observations on FC supplementation after spinal cord compression.^20^

The multi-nutrient, FC, was developed to prevent destabilization and loss of synapses, an early feature of AD,^119^ and several studies support this mechanism of action.^36^ In our study, the presynaptic protein, synaptophysin, and the post-synaptic protein, PSD-95, were used to assess synapse loss post-TBI. In the FC group, we saw a total reversal in the decrease in synaptophysin induced by the injury, and a similar trend was observed for PSD-95.

Taken together, the data show pleiotropic beneficial effects of supplementation with PL precursors post-TBI. The functional improvement using neurological endpoints is correlated with improved tissue aspect, ipsilaterally and also in the contralateral hemisphere (which has an important role in recovery post-injury).^116^

PC and PE levels were reduced post-CCI even at a distance from the injury site, as reported previously,^18,19^ and FC reduced these losses.

The multi-nutrient used in this study provides precursors for the formation of PLs,^31^ and this novel therapeutic strategy addresses mechanistically the chronic decrease in PL detected post-TBI in humans.^22^ This consequence of injury is persistent, that is, detectable at 24 months post-trauma, in an impact brain injury model in mice,^120^ suggesting that it is a fundamental process of destabilization of membranes, observed across species. Stocchetti and colleagues have proposed a list of key requirements for treatments for TBI tested pre-clinically, in order to increase the chances of successful clinical translation.^121^ The PL precursor combination tested here satisfies several requirements: 1) the relevance of the targeted mechanism (i.e., there is a reported sustained decrease of 25–35% in several PL classes post-injury after TBI; 2) the treatment tested can increase brain PL levels after 4–6 weeks of supplementation (as shown by Cansev and colleagues in aged rats^122^ and our data here); and 3) the PL precursor preparation has been previously shown to restore brain connectivity and reduce hippocampal loss in patients, in early AD, as supported by clinical trials with FC.^34,123,124^

TBI triggers many injury mechanisms, which should be addressed using multi-modal treatments.^125^ Specialized nutrient interventions have the benefit of addressing multi-modal mechanisms,^126^ and FC is a unique concept focused on the support of membrane integrity through provision of precursors required for PL formation. Injury in the nervous system triggers an extensive reorganization of circuits ipsilaterally and in the opposite hemisphere, and it could be hypothesized that FC might provide support for such restorative processes.^116,127–129^

Our results show that the novel concept of providing PL precursors post-injury could have therapeutic potential in TBI. Future studies should explore in detail in several TBI models the time window for administration and the optimum duration of intervention, in order to increase the likelihood of translational success.

## Supplementary Material

Supplemental data

Supplemental data

Supplemental data

Supplemental data

Supplemental data
