# Supplementary material for: Brain Phospholipid Precursors Administered Post-Injury Reduce Tissue Damage and Improve Neurological Outcome in Experimental Traumatic Brain Injury
Source: J Neurotrauma. 2018 Dec 14;36(1):25–42. doi: 10.1089/neu.2017.5579 (PMC6306688; doi:10.1089/neu.2017.5579)
Supplement: Supplemental data [file Supp_Fig2.pdf]

Task	Score
**Exit-time***	1-3
**Walking straight**	1
**Startle reflex**	1
**Seeking behaviour**	1
**Hemiparesis**	1
**Round stick balance**	1
**Triangle stick balance**	1
**Beam walking****	
**1cm**	1-3
**2cm**	1-3
**3cm**	1-3

*Exit task-points	Score
exit within 20sec	0
exit within 60sec	1
exit within 2min	2
no exit within 2min	3
**Beamwalk-points	
Balance and walk with normal posture	0
Grasp side of the beam+ draging OR sliping poterior paw less than 3 times	1
more than 3 times and no abiliti togrip	2
unable to walk the beam	3

SUPPLEMENTARY FIG. S2. Modified neurological severity score test (mNSS)—table of content Modified Neurological Severity Scores (NSS) outlined in Table. This modified test consists of 10 individual clinical parameters, including tasks on motor function, alertness, and physiological behavior, to evaluate neurological impairment. One point is awarded for the inability to perform the tasks. A maximal NSS of 18 points thus indicates severe neurological dysfunction, with failure of all tasks. Modifications of the scoring are represented with asterisks and detailed respectively in the second table.
