# Supplementary material for: Brain Phospholipid Precursors Administered Post-Injury Reduce Tissue Damage and Improve Neurological Outcome in Experimental Traumatic Brain Injury
Source: J Neurotrauma. 2018 Dec 14;36(1):25–42. doi: 10.1089/neu.2017.5579 (PMC6306688; doi:10.1089/neu.2017.5579)
Supplement: Supplemental data [file Supp_Fig3.pdf]

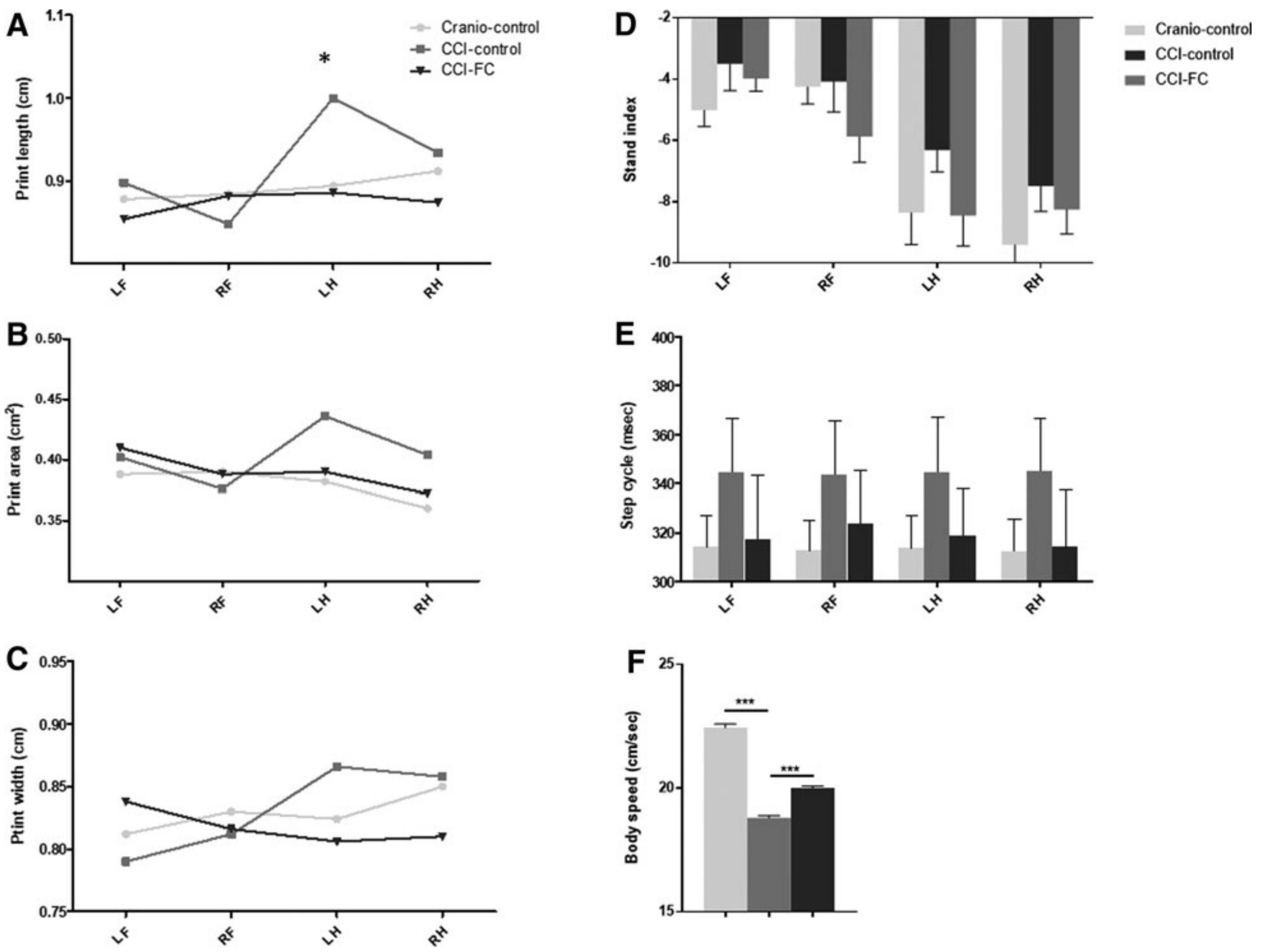

SUPPLEMENTARY FIG. S3. Gait impairment assessment after TBI was assessed at 2 dpi, using the CatWalk XT system. Behavior data were analyzed using two-way ANOVA analysis. The FC-treated group showed a decrease in (**A**) print length (horizontal direction; LH, $*p < 0.05$ compared to CCI-control) on the side contralateral to the injury, with an emphasis on the left hindpaw. A longer print length could reflect foot dragging and overall less control of the foot. We also noticed a clear tendency to a smaller (**B**) print width and (**C**) print area (vertical direction) in the CCI-control group (ns; two-way ANOVA, $**p < 0.01$). The stand index (SI) was higher in CCI-control animals compared to the two other groups ($***p < 0.001$; **D**). The overall step cycle (**E**) was longer and the body speed (**F**), significantly slower in the CCI-control group compared to CCI-FC and craniotomy-control (ns; two-way ANOVA, $*p < 0.05$ subjects, Bonferroni's post-hoc test, $***p < 0.0001$, respectively. Data are means ± SEM of 10 animals/group. ANOVA, analysis of variance; CCI, controlled cortical impact; dpi, days post-injury; FC, Fortasyn® Connect; LF, left front; LH, left hind; ns, not significant; RF, right front; RH, right hind; SEM, standard error of the mean; TBI, traumatic brain injury.
