# Supplementary material for: Brain Phospholipid Precursors Administered Post-Injury Reduce Tissue Damage and Improve Neurological Outcome in Experimental Traumatic Brain Injury
Source: J Neurotrauma. 2018 Dec 14;36(1):25–42. doi: 10.1089/neu.2017.5579 (PMC6306688; doi:10.1089/neu.2017.5579)
Supplement: Supplemental data [file Supp_Fig4.pdf]

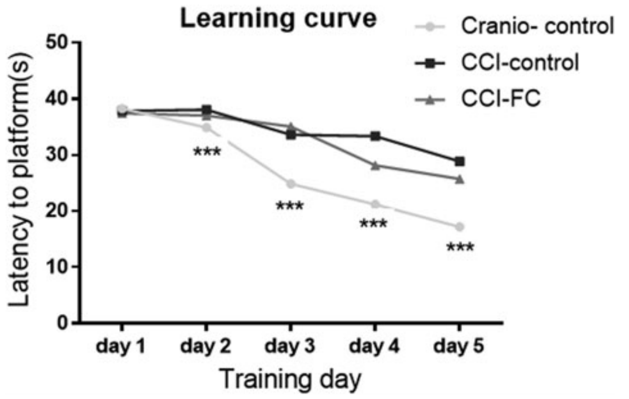

SUPPLEMENTARY FIG. S4. Evaluation of learning acquisition curve in MWM test. Five consecutive days of acquisition training sessions were assessed on 13–17 dpi. Mean latencies to find the platform were recorded. Two-way ANOVA, ***$p < 0.001$, Bonferroni's post-hoc test: day 2, Cranio-control versus CCI-control and CCI-FC, ***$p < 0.001$; day 3, Cranio-control versus CCI-control and CCI-FC, ***$p < 0.001$; day 4, all group comparison, ***$p < 0.001$; day 5, cranio-control versus CCI-control, ***$p < 0.001$ and cranio-control versus CCI-FC, *$p < 0.05$. Data are means ± SEM of 10 animals/group. ANOVA, analysis of variance; CCI, controlled cortical impact; dpi, days post-injury; FC, Fortasyn® Connect; MWM, Morris water maze; SEM, standard error of the mean.
