# Supplementary material for: Brain Phospholipid Precursors Administered Post-Injury Reduce Tissue Damage and Improve Neurological Outcome in Experimental Traumatic Brain Injury
Source: J Neurotrauma. 2018 Dec 14;36(1):25–42. doi: 10.1089/neu.2017.5579 (PMC6306688; doi:10.1089/neu.2017.5579)
Supplement: Supplemental data [file Supp_Fig5.pdf]

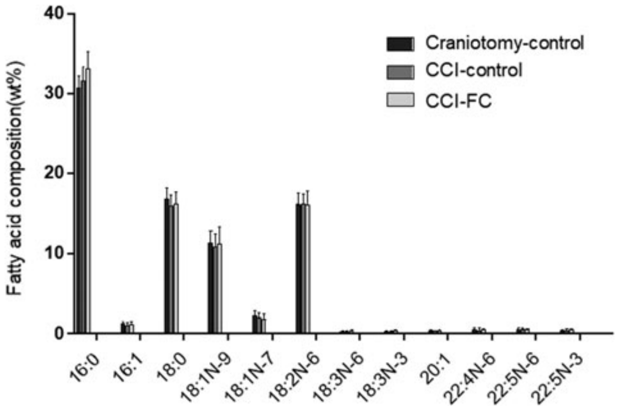

SUPPLEMENTARY FIG. S5. Other fatty acid composition. The total phospholipid fatty acid content of the plasma samples was determined. Data are means ± SEM of 10 animals/group. CCI, controlled cortical impact; FC, Fortasyn® Connect; SEM, standard error of the mean.
